# *In silico *and *in vitro *comparative analysis to select, validate and test SNPs for human identification

**DOI:** 10.1186/1471-2164-8-457

**Published:** 2007-12-12

**Authors:** Emiliano Giardina, Ilenia Pietrangeli, Claudia Martone, Paola Asili, Irene Predazzi, Patrizio Marsala, Luciano Gabriele, Claudio Pipolo, Omero Ricci, Gianluca Solla, Luca Sineo, Aldo Spinella, Giuseppe Novelli

**Affiliations:** 1 Centre of Excellence for Genomic Risk Assessment in Multifactorial and Complex Diseases, School of Medicine, Tor Vergata University of Rome, Italy; 2 Direzione Centrale Anticrimine, Servizio di Polizia Scientifica, Rome, Italy; 3 Dipartment of Animal Biology, University of Palermo, Italy; 4 Division of Cardiovascular Medicine, Department of Medicine, University of Arkansas for Medical Sciences, Little Rock, AR 72205, USA

## Abstract

**Background:**

The recent advances in human genetics have recently provided new insights into phenotypic variation and genome variability. Current forensic DNA techniques involve the search for genetic similarities and differences between biological samples. Consequently the selection of ideal genomic biomarkers for human identification is crucial in order to ensure the highest stability and reproducibility of results.

**Results:**

In the present study, we selected and validated 24 SNPs which are useful in human identification in 1,040 unrelated samples originating from three different populations (Italian, Benin Gulf and Mongolian). A Rigorous *in silico *selection of these markers provided a list of SNPs with very constant frequencies across the populations tested as demonstrated by the F_st _values. Furthermore, these SNPs also showed a high specificity for the human genome (only 5 SNPs gave positive results when amplified in non-human DNA).

**Conclusion:**

Comparison between *in silico *and *in vitro *analysis showed that current SNPs databases can efficiently improve and facilitate the selection of markers because most of the analyses performed (F_st_, r^2^, heterozigosity) in more than 1,000 samples confirmed available population data.

## Background

Genetic variation in the human genome takes many forms, ranging from large, microscopically visible chromosome anomalies to single-nucleotide changes. In general, forensic DNA analysis compares biological samples searching for genetic similarities and differences by typing a small number of genetic variable segments in each sample. Current methods used for performing forensic DNA analysis mainly focus on typing several STR (Short Tandem Repeats) markers. STRs are smaller than VNTR sequences and can easily discriminate between both related and unrelated individuals [1]. At present, different optimized multiplex assays are used to analyze unique STRs loci located on different chromosomes with the advantage of providing extremely low random match probability (the probability of finding the same DNA profile in a randomly selected, unrelated individual). The only drawback in current forensic DNA typing systems is that sometimes PCR amplicons are excessively large (ranging from 100–400 bases in length). Sometimes DNA samples appear degraded into smaller fragments and therefore, the higher molecular-weight STR loci can be scarcely amplified so creating incomplete DNA profiles with lower discrimination power [2]. The presence of alternative types of DNA polymorphic markers in the human genome i.e. Single Nucleotide Polymorphisms (SNPs), abundantly spread along the chromosomes, make it possible to develop different DNA profiling techniques able to type smaller fragments of DNA (lower than 100 bp) compared to those detectable with STR markers (100–400 bp). In addition, due to the biallelic nature of SNPs, a size-based separation is not necessary so in turn making a higher level of multiplexing and automation possible compared to when using STRs. The latter are extremely important in the implementation of large criminal DNA databases [3,4]. Furthermore, SNPs have smaller mutation rate (about 10^-8^) [5] compared to STRs (ranging from 10^-3 ^to 10^-5^) [6,7] and the number of chromosomes which need typing to assess allele frequencies for SNPs are lower compared to STRs because of the smaller number of alleles. Nonetheless, several important factors have to be considered for an accurate selection of the SNPs to be used in forensic analysis. The first issue concerns the correct evaluation of the frequency of each SNP among the populations. STRs markers have many alleles and each of them show worldwide low frequency and consequently the random match probabilities are not usually strongly dissimilar among the populations. Conversely, SNP markers can show very dissimilar frequencies among different populations, causing a very large dependence of the match probability on the population frequencies used for the calculation [6]. An incorrect evaluation of SNPs frequencies may give rise to ambiguity in genetic results under specific conditions or in isolated populations. A second issue originates from the necessity to facilitate the stability and reproducibility of genetic typing for which forensic SNPs should be exclusively found in the human sequence and mapped in a single locus (single copy SNP). Another important issue to consider for a correct SNPs selection follows the discovery reported in several recent studies regarding the presence of an abundance of submicroscopic copy number variation of DNA segments ranging from kilobases (kb) to megabases (Mb) which include deletions, insertions, duplications and complex multi-site variants in all humans and other mammals [8]. These number variable regions (CNVRs) cover 12% of the genome. It is thus preferable that SNPs used in human identification are located outside the copy number variable regions. In addition, the SNPs used in forensic analysis should referentially not be located in coding/regulatory regions in order to avoid the possibility of obtaining unnecessary information concerning health status and/or disease susceptibility or resistance of the individual analyzed thus giving the courtroom the possibility to reject DNA tests [9]. It follows that forensic markers should not be published as susceptibility/resistance/prognostic factors in scientific publications. The above mentioned issues highlight the importance of the availability of genetic databases to help facilitate SNPs selection. In this work, we present a combination of *in silico *and *in vitro *genetic analysis to validate and test a panel of 24 SNPs for human identification, selected on the basis of very stringent criteria. A total of 24,960 genotypes of individuals originating from three different continents (Europe, Africa and Asia) were analysed to assess allele frequencies and the results were compared with current available SNPs databases. The specificity of selected SNPs for the human genome was tested by combining *in silico *selection and *in vitro *testing of non-human DNA. In this study, we provide a list of 24 SNPs specifically designed for human identification and recommend an intensive use of current genetic database which can strongly improve the *a priori *selection of the SNPs because most analyses performed (F_st_, r^2^, heterozigosity) in our samples confirmed *in silico *analysis.

## Results

Selected SNPs are distributed across 13 autosomes and the two sexual chromosomes. Six chromosomes contain more than 1 SNP. The frequencies of the chosen SNPs are reported in Table 1. Genotype frequencies observed for the selected SNPs in 1,040 unrelated individuals showed their high informative capacity thus confirming the data reported in the HapMap database. The heterozygosity values calculated in the three populations considered in this study range between 0.410 and 0.497, the mean value being 0.473 with a standard deviation of 0.01. These latter values are quite similar to those reported in the HapMap database. In particular, the average heterozygosity observed in the typed Italian samples was 0.460 compared to the 0.480 calculated on the basis of genotype frequencies reported for CEU samples in the HapMap database. No differences were noted between the heterozygosity observed in the Benin and Mongolian samples (0.480 and 0.480 respectively) and those reported for YRI and CHB in the HapMap database (0.486 and 0.486 respectively). While no differences in the mean heterozygosity of the entire panel of SNPs were observed, significant differences in allele frequencies of a number of SNPs compared to those reported in the HapMap database were noted. We observed significant differences between the Italian and CEU (CEPH- HapMap project) samples for four SNPs: rs905213 (p = 0.001), rs1533800 (p = 0.009), rs886528 (p = 0.047), rs154659 (0.028) (Table 1). It should be mentioned that CEPH samples were collected from people living in Utah with ancestry from northern and western Europe collected in 1980 and it is unclear how accurately these samples reflect the patterns of genetic variation in people with northern and western European ancestry. We also observed a highly significant difference in allele frequency between the Benin and Yoruba samples (HapMap Project) for the SNP rs3130315 (p = 2 × 10^-5^) and between the Mongolian and Han Chinese samples (HapMap project) for two SNPs: rs9562080 (p = 0.047) and rs4116821 (7.36 × 10^-11^). Considering that genetic markers may also show dissimilar allele frequencies among populations due to differences in mutation rates, neutrality or linkage disequilibrium with other loci subjected to selection, the F_st _distribution of the selected SNPs in these populations was calculated. For each autosomal marker, the lower the F_st _value among the populations, the higher is the worldwide applicability of that specific SNP for human identification. F_st _values ranged between 0.001 and 0.143, while the mean value observed for all the autosomal SNPs was 0.037 ± 0.047. Calculation of F_st _values on the HapMap data generated similar results: F_st _values ranged between 0.0000 and 0.0792, while the mean value observed for all the autosomic SNPs is 0.028 ± 0.025. In order to ensure the independence of markers, all the syntenic autosomal SNPs selected were very far from each other, with a minimum distance of 27 Mb observed between rs1506981 and rs1533800 on chromosome 11 (Table 1). Linkage disequilibrium (LD) decays with map distance along the chromosome at a rate determined by the effective population size and therefore should not be expected in our syntenic SNPs. However, we assessed the independence of variation for all the selected SNPs by calculating pairwise LD values reported as r^2^. Most of the selected autosomal SNPs showed r^2 ^values near zero: the average was 0.01 ± 0.02 in Europeans; 0.02 ± 0.07 in Africans; 0.07 ± 0.09 in Asians. The use of LD plotter software [10] revealed no significant evidence of linkage disequilibrium (LD) between markers mapped on chromosome Y (rs4116821, rs1421177 and rs2032652) [11]. All the SNPs were selected to be specific for a single locus in the human genome. The typing of the SNPs in the DNA from the closest living relative of Homo sapiens (*Pan troglodytes*) and *Macaca fascicularis *made it possible to confirm in silico specificity (tested by Blast) for 19 SNPs. We observed positive amplification for a single marker in *Macaca fascicularis *and for five markers in *Pan troglodytes*. SNP rs886528 was typed in *Macaca fascicularis *DNA and SNPs rs1779866, rs905213, rs1506981, rs8033863 and rs886528 were typed in *Pan troglodytes *DNA. All TaqMan assays that provided interpretable signal in real-time PCR were re-sequenced to determine the exact sequence homologies between the species. As expected on the basis of *in silico *and *in vitro *results, the random match probability using the entire 24 SNPs panel was very similar in the three populations tested: 5.7 × 10^-11 ^in Europeans, 7.8 × 10^-10 ^in Africans, 3.4 × 10^-11^. The cumulative random match probability shown above indicates that the likelihood that a specific profile occurs in more than a single individual is less than 1 in 100,000,000,000.

**Table 1:** Chromosome localization, nucleotide position, allele variations, average heterozygosity and F_st _values of the selected markers. Minor allele frequency (MAF) of HapMap data are reported in the brackets.

					**European Population**	**African Population**	**Asian Population**	
**SNPs**	**Chr**	**Nucleotide position**	**Variation**	**Hap map Heterozygosity**	**Minor all. frequency**	**Minor all. frequency**	**Minor all. frequency**	**F_st_**

**rs1779866**	1	61,194,420	C/G	0.497	0.43 (0.44)	0.47 (0.50)	0.45 (0.50)	0.0126
**rs1922807**	2	77,574,919	A/G	0.493	0.46 (0.50)	0.37 (0.40)	0.38 (0.46)	0.0318
**rs2278741**	2	121,465,927	C/G	0.470	0.27 (0.31)	0.37 (0.40)	0.40 (0.53)	0.1430
**rs2962594**	5	2,759,621	A/G	0.477	0.38 (037)	0.44 (0.43)	0.45 (0.37)	0.0333
**rs905213**	5	91,995,945	C/T	0.463	**0.38 (0.61)**	0.35 (0.35)	0.41 (0.36)	0.0000
**rs11242909**	6	422,065	C/T	0.536	0.33 (0.30)	0.38 (0.50)	0.39 (0.50)	0.0000
**rs3130315**	6	32,328,663	A/G	0.467	0.38 (0.38)	**0.37 (0.67)**	0.46 (0.50)	0.0634
**rs7740233**	6	150,965,880	C/T	0.477	0.43 (0.44)	0.45 (0.42)	0.44 (0.51)	0.0136
**rs10866988**	8	5,235,745	C/G	0.500	0.47 (0.46)	0.49 (0.50)	0.40 (0.43)	0.0127
**rs1506981**	11	6,735,647	A/G	0.487	0.55 (0.53)	0.33 (0.37)	0.34 (0.44)	0.0146
**rs1533800**	11	33,781,074	A/C	0.487	**0.22 (0.49)**	0.42 (0.45)	0.41 (0.42)	0.1278
**rs1981752**	12	16,430,035	C/T	0.487	0.33 (0.36)	0.44 (0.48)	0.49 (0.52)	0.0955
**rs478347**	12	128,915,904	G/T	0.510	0.49 (0.48)	0.46 (0.45)	0.42 (0.35)	0.0032
**rs9562080**	13	113,941,879	C/G	0.483	0.50 (0.39)	0.44 (0.45)	**0.47 (0.61)**	0.0034
**rs8033863**	15	99,857,192	C/G	0.407	0.37 (0.38)	0.36 (0.43)	0.40 (0.39)	0.0000
**rs886528**	16	3,751,557	C/T	0.500	**0.38 (0.52)**	0.40 (0.47)	0.45 (0.43)	0.0450
**rs154659**	16	88,194,838	C/T	0.467	**0.16 (0.29)**	0.49 (0.46)	0.34 (0.42)	0.1229
**rs2317225**	17	2,593,098	A/G	0.490	0.37 (0.38)	0.45 (0.52)	0.39 (0.48)	0.0029
**rs11881170**	19	62,864,113	A/C	0.497	0.39 (0.44)	0.41 (0.42)	0.35 (0.30)	0.0000
**rs380011**	21	14,542,669	C/T	0.487	0.39 (0.36)	0.48 (0.48)	0.45 (0.50)	0.0133
**rs2267628**	X	137,456,866	A/G	-	0.49 (0.46)	0.03 (0.02)	0.21 (0.12)	-
**rs4116821**	Y	20,531,728	C/G	-	0.37 (0.46)	0.01 (0.19)	**0.35 (0.00)**	-
**rs1421177**	Y	18,027,988	G/A	-	0.00	0.00	0.00	-
**rs2032652**	Y	20,305,438	C/T	-	0.13 (0.00)	0.00 (0.00)	0.46 (0.46)	-

## Discussion

We carried out a study regarding the selection of a panel of 24 SNPs for forensic analysis. We mainly focused on *in silico *selection of SNPs to avoid unspecific non human amplification and unbalanced allele frequencies among the populations. Through *in silico *searches, we selected SNPs located in only human-specific sequences. In vitro analysis showed that only five SNPs gave positive results when amplified in DNA from the closest living relative of Homo sapiens (*Pan troglodytes*) and from *Macaca fascicularis*. We would like to emphasise that this number is lower than figures reported do date in other studies. The latter demonstrates how useful it is to carefully select SNPs by comparing genomic sequences of different species [12-15]. We also excluded an interdependence of selected markers (to both autosomes and Y chromosome) by calculating the extention of LD (r^2 ^values were not significant).

When considering the average values (heterozygosity; allele frequencies, F_st _values, RMP) of the entire panel of SNPs calculated on the HapMap data, we observed no significant deviation. Thus, the availability of genetic databases strongly facilitates SNPs selection as to their informative capacity: a priori selection criteria were confirmed by the genotyping of our populations. In the present study, we report SNPs showing very high heterozygosity values: the mean was 0.473 ± 0.01. Although it is possible to observe significant differences between allele frequencies for specific SNPs, on average, the deviation in allele frequencies of a panel of SNPs between "expected" (HapMap) and "observed" (genotyping) seems not to be significant. These results may be due to divergences among the populations analyzed in this work and those typed in HapMap, or from the limited size of samples analyzed in the HapMap project. In fact, the mean heterozygosity calculated on the basis of genotype frequencies reported in the HapMap database was quite similar to the one calculated in this work (0.484 ± 0.04). there is a similar situation regarding differences in F_st _values. Accordingly, the differences in random match probabilities calculated on the basis of both observed and expected frequencies are not relevant. In particular, the RMP arising from data (CEU) reported in HapMap was 2.4 × 10^-11 ^quite similar to the one calculated in this work 5.7 × 10^-11 ^(Italian). The same situation is evident in African and Asian populations where the RMP observed were similar to those calculated with the HapMap data (in the brackets): 7.8 × 10^-10 ^(8.1 × 10^-10^) in Africans; 3.4 × 10^-11 ^(2.4 × 10^-10^). These data suggest that the main effort in developing a SNP panel for forensic purposes should be mainly directed towards the selection of universal forensic SNPs to be used and on typing admixed populations in order to confirm the worldwide balance of allele frequencies of SNPs. The efficiency of the programs used for the alignment of genome sequences can strongly improve the process of selection of human-specific SNPs.

## Conclusion

In this paper, we selected and validated 24 SNPs useful for human identification. To generate allele frequencies, we used the Real-Time PCR. However, it should be noted that this technique cannot produce a multiplexing of the PCR and therefore, it could be of only limited use in forensic practice. We showed that current SNPs databases can efficiently improve and facilitate the selection of markers since most of the reported frequencies have been confirmed in recent works. The most important aspect of SNPs selection criteria is the choice of those SNPs with low F_st _among populations and with a single locus location exclusively in the human genome. Rigorous marker selection criteria have been fundamental for performing a SNP-base human identification. Compared to previous reported SNPs selection [16], here we report a much higher percentage of SNPs (80%) specific for the human genome. We also recommend selecting markers located outside regions containing copy number variations. So far, the availability of several maps of copy number variations in the human genome has shown that we are genetically more diverse than expected and that our genome appears to be even more amorphous and changeable than expected. Consequently, the selection of SNPs for human identification needs to consider all these variables in order to ensure the highest stability and reproducibility of results. During the final phases of this work, a CNV map of the human genome was published through the study of 270 individuals from four of the populations typed in the HapMap project. Three new segmental duplications were observed within the RP11-420I5, RP11-530H6 and RP11-469G12 clones which contain respectively selected markers rs478347, rs8033863 and rs2317225. It is important to underline, however, that forensic markers (WVA and FGA) in current use are also located in CNV regions but no genotyping errors have been reported to date and today we do not know how many genomic regions may have CNVs because the variability of the human genome is much higher than previously supposed [17] and new CNV regions are constantly being reported. Despite their informative capacity and the absence of typing errors observed in this work, the use of the SNPs rs478347, rs8033863 and rs2317225 as markers for forensic purposes should be considered with caution due to the fact that typing errors arising from the complexity of the surrounding genomic regions cannot be totally excluded.

## Methods

### Samples and DNA extraction

A total of 1,040 samples from Italy, the Benin gulf and Mongolia were collected. The QIAamp DNA Blood Mini Kit (QIAGEN Inc., Valencia, CA) was used to extract genomic DNA from whole blood. DNA concentration was determined by real-time PCR using the Quantifiler™ Human DNA Quantification Kit (Applied Biosystems).

### Selection Criteria

A number of selection criteria were used to identify the SNPs considered in this work (Table 1):

(i) SNPs location outside coding regions; not published as resistance/susceptibility allele;

(ii) average minimal allele frequency (MAF) reported in population databases of at least 0.4 by searching in population databases [18].

(iii) SNPs location in a single locus in the human genome;

(iv) SNPs specificity for the predicted locus evaluated by comparison of the sequences flanking the selected markers to those available in the Genebank database. A heuristic search using the Genome BLAST (blastn) option was performed using default parameters. BLAST (Basic Local Alignment Search Tool) is a sequence similarity search program that finds matches between a tag sequence and human and non human sequences available in databases [19]. SNPs whose surrounding sequence was found in different sites other than the one predicted, or which showed too much similarity to sequences belonging to other species' genomes, were not considered candidate markers for human identification.

(v) examination of the flanking regions of the SNPs to ensure that regions surrounding the polymorphic site contained no additional variations. For two selected SNPs (rs1922807 and rs886528), potential interfering additional polymorphisms at a distance of 46 bp upstream and 40 bp downstream respectively were present but they are not relevant in most genetic typing;

(vi) SNPs location outside the copy number variation regions by search in the Database of Genomic Variants [20]. In the final phase of this work, novel CNVs were published encompassing regions involving three selected SNPs rs478347; rs8033863; rs2317225 [8]. The three segmental duplications within the RP11-420I5, RP11-530H6 and rp11-469g12. clones contain respectively SNPs rs478347, rs8033863 and rs2317225.

### Genotyping

Genotyping was performed by TaqMan assays (Applied Biosystems). Primers and probe sequences of each Taqman assay are listed in Table 2. The average size of the amplicons was 77 bp. Reactions were run in an AB7500 (Applied Biosystems) and interpreted using Sequence Detection System (SDS) 2.1 software. Each plate contained three positive controls (samples previously confirmed by direct sequencing as heterozygous and both homozygous) and two negative controls. No departure from the Hardy-Weinberg equilibrium was detected. Genotype assessment for each SNP was confirmed by post-genotyping direct re-sequencing of 50 random samples.

**Table 2:** Primers and probes sequences of the SNPs tested.

**SNP name**	**PRIMER Forward**	**PRIMER Reverse**	**PROBE 1**	**PROBE 2**
**rs1779866**	5'-GGGATTAACTTTGCAAATGGAATTAAAGGA-3'	5'-CCCTTTTAAGGACACTTTTGATTGCTT-3'	5'-CATAATCGAAGATCCTC-3'	5'-CATAATCCAAGATCCTC-3'
**rs1922807**	5'-CATAGTCCCATAAGAATATGCAGCATGT-3'	5'-GTTGTAAGAAAACATATTAAAAGCGGTACCA-3'	5'-CAATAGATGATTCAATAATTT-3'	5'-AATAGATGATTCAGTAATTT-3'
**rs2278741**	5'-CCGTGTTTACAGTGTTTGCATGT-3'	5'-AGCAGCCCCGAGGTATTTAGA-3'	5'-CCTGAAAAGAACACTTGTT-3'	5'-CTGAAAAGAAGACTTGTT-3'
**rs2962594**	5'-GTCTTCCCCTCCCTTCTTTGTC-3'	5'-GAAGTGTTATCAAAATACACCTTGGAAATATCA-3'	5'-ACATGGTCAGCACTGG-3'	5'-CATGGTCAGCGCTGG-3'
**rs905213**	5'-GGCTGATAACGTAACCCGAACT-3'	5'-ATGAAGATCGGTGGCGGAAAT-3'	5'-CAATTTAATAAGTCTGAAAGAC-3'	5'-AATTTAATAAGTCTAAAAGAC-3'
**rs11242909**	5'-TGCCATCAAGTATATTTTGTGTGATATCAACT-3'	5'-CCCCAAAAAAAATGTTTGCTAGGGAAA-3'	5'-AAAAGAAAGCTAGTGGTGCTA-3'	5'-AAAAGAAAGCTAATGGTGCTA-3'
**rs3130315**	5'-AGCACTAGAGCTGCAAAGATATTGG-3'	5'-GTCCCTTCTGAAACCTTACACATCA-3'	5'-CATCTCACTCTCTAACCAA-3'	5'-CTCACTCTCCAACCAA-3'
**rs7740233**	5'-GAGAGCCCCTTCTGCTTGTTT-3'	5'-AGCTCCTTGCTATTCTCAATTCTTATAAAACTC-3'	5'-CCTTTGTCTCAGGTCCTA-3'	5'-CCTTTGTCTCAAGTCCTA-3'
**rs10866988**	5'-GCATCGGAAGCCATATTTTGTGT-3'	5'-ATTGTTTGACTCTTTTGGCCGTTTT-3'	5'-CTTCTCCCAAGGCCA-3'	5'-CTTCTCCGAAGGCCA-3'
**rs1506981**	5'-TCGGCTATGGAGGCCTATAAGAG-3'	5'-CAAAGATGCCTTGAGTTTTTATGTGATTTAGA-3'	5'-CCACCAGGTTTCA-3'	5'-CCACCGGGTTTCA-3'
**rs1533800**	5'-CCTGAGGAGGCAGTGTGG-3'	5'-AGACTAGGTCAGCAGGAACTCA-3'	5'-CAGATCCCAGATCTAGT-3'	5'-CAGATCCCAGCTCTAGT-3'
**rs1981752**	5'-CTGCAAATGCTAATTGATAGATCAGCAA-3'	5'-GGTAGAAAAGACGCAAAACTCCAAA-3'	5'-AAACTGTATGTGTTAAAAG-3'	5'-AAACTGTATGTATTAAAAG-3'
**rs478347**	5'-CCCATCTTTAGAGGGCCTGAAA-3'	5'-GCAGAAAGGAGCTGGAATTCAAGTA-3'	5'-TCCACCAGTGGCATAG-3'	5'-ATCCACCAGTTGCATAG-3'
**rs9562080**	5'-CCACCATTGATCTCTGAGCATCTC-3'	5'-AGGGTGTGGGCGGTG	5'-TGGCTGGGCCCCT-3'	5'-TGGCTGGGCGCCT-3'
**rs8033863**	5'-GGAAGGAGTTGGCTAATACAATGCT-3'	5'-CCAGGGCTCTAACACATATTTTAAAAATCA-3'	5'-ACAGCGCTGCCACC-3'	5'-ACAGCCCTGCCACC-3'
**rs886528**	5'-GAGGTTGGTCAAGAGTCTGTATCC-3'	5'-CAGGCGCACCAAGAACATG-3'	5'-ATTTCCATCAGAGCCCA-3'	5'-TTTCCATCAAAGCCCA-3'
**rs154659**	5'-CCCCTCCTGACCTCGAATTAGA-3'	5'-CACATCCAGGCAGGTCATACC-3'	5'-CAGGCTATTCTCGGCAAG-3'	5'-CAGGCTATTCTCAGCAAG-3'
**rs2317225**	5'-TCACCCAAGTCCAAGCCAAAA-3'	5'-AGATGACAGTCAGGAGCCTACT-3'	5'-CTTCTGGTGTACCATTG-3'	5'-TCTGGTGTGCCATTG-3'
**rs11881170**	5'-TGTTGACTAGAGGCTGTGAG-3'	5'-TCATGCCCAC ACTATGTCTA-3'	5'-TTGAGCCTTCCTTACTAGTTACTA-3'	5'-TTGAGCCTTCATTACTAGTTACTA-3'
**rs380011**	5'-TGAGGAATGAGTTTATGTCCATTGAGTT-3'	5'-CCACCCATAGCCCTCTGATTC-3'	5'-CAGGTAAGAATAGGATTT-3'	5'-CAGGTAAGAATAAGATTT-3'
**rs2267628**	5'-TCTTGGGGAAGGAGTGTTGT-3'	5'-TCAATGTGAGCGCTAAGACT-3'	5'-TTCATGAATAGATGATGCAACT-3'	5'-TTCATGAATAAATGATGCAACTT-3'
**rs4116821**	5'-CCATTATGCCTTTCTTTGAG-3'	5'-TTATGTACAAAAATCTCATCTCTCACTTTG-3'	5'-GAGCTACGAGATGTAAAAGGGA-3'	5'-GAGCTACCAGATGTAAAAGGGA-3'
**rs1421177**	5'-TTGCCCTGTATGCCTACAAC-3'	5'-TTGCCAGAGACTAGAAAGAA-3'	5'-TTAGCTAACTCAAAACTCACAG-3'	5'-TTAGCTAACTCAGAACTCACAG-3'
**rs2032652**	5'-TTGGCCCAACACTGTTGCCA-3'	5'-TTTGCTGGTGATCCTGGACC-3'	5'-TATGTACAAAAATCTTATCTCTCACT-3'	5'-TATGTACAAAAATCTCATCTCTCACT-3'

### Statistical analysis

All the statistical analyses of forensic data were performed as described by Brenner [9] and using DNAVIEW™ 27.19. Statistical independence of selected markers was assessed by calculating linkage disequilibrium (LD) as r^2 ^[10] using LDplotter software. Inter-population variability in allele frequencies was assessed by calculating F_st _for each marker [21] To test the null hypothesis of no differences in allele frequencies between the HapMap and typed populations, the frequency distribution of alleles was analysed for our samples as well as for the HapMap samples, using X^2 ^test.

## Authors' contributions

EG conceived the study and participated in its design and in the interpretation of the data;

IPi, CM, carried out the molecular genetic studies and statistical analysis;

PA, participated in the design of the study;

IPr, carried out the *in silico *analysis;

PM, LG, CP, OR, GS carried out the DNA extraction and sample quantification;

LS, was involved in drafting the manuscript and in the non-human DNA analysis;

AS, GN, made useful critical comments and coordinated the research group.

All authors read and approved the final manuscript.
